# Effect of Sb on the Hot Ductility and Fracture Behavior of Low-Alloy Corrosion-Resistant Steel

**DOI:** 10.3390/ma19112202

**Published:** 2026-05-23

**Authors:** Zhiwei Liu, Wang Li, Xiuhua Gao, Linxiu Du, Hongyan Wu, Ruiqi Zhang

**Affiliations:** 1 State Key Laboratory of Metal Material for Marine Equipment and Application, Anshan 114009, China; 2 Iron and Steel Research Institute of Angang Group, Anshan 114009, China; 3 State Key Laboratory of Digital Steel, Northeastern University, Shenyang 110819, Chinadulx@ral.neu.edu.cn (L.D.); wuhy@ral.neu.edu.cn (H.W.)

**Keywords:** corrosion-resistant steel, hot ductility, Sb, ductility trough, intergranular fracture

## Abstract

The mechanism by which Sb influences the hot ductility and fracture behavior of corrosion-resistant steel within the temperature range of 650–1200 °C was systematically investigated using scanning electron microscopy (SEM) and electron probe microanalysis (EPMA). The temperature interval of the ductility trough and the underlying mechanisms responsible for its occurrence were elucidated. The results indicated that ductility troughs for the 0.09Sb and 0.15Sb steels occurred at 726–949 °C and 736–995 °C, respectively. Increasing Sb content broadened the ductility trough temperature range and shifted the minimum ductility temperature to higher values. The ductility trough was attributed to the combined effects of grain boundary ferrite films, coarse precipitates, and non-equilibrium grain boundary segregation of Sb. During deformation in the austenite–ferrite two-phase region at 800 °C, the hot ductility is primarily governed by the thickness of the grain boundary ferrite film. These ferrite films are prone to stress concentration, thereby reducing the hot ductility of both the 0.09Sb steel and the 0.15Sb steel. In the single-phase austenite region at 900 °C, coarse Ti(C,N) and MnS precipitates readily act as crack initiation sites, leading to intergranular fracture in the 0.15Sb steel. Non-equilibrium Sb grain boundary segregation further weakens grain boundary cohesion, thereby deteriorating the hot ductility of the steel. Moreover, increasing Sb content enhanced the magnitude of non-equilibrium grain boundary segregation and elevated its peak temperature, thereby raising the minimum ductility temperature. This work provides a theoretical basis and technical guidance for optimizing the continuous casting of Sb-containing corrosion-resistant steel in industrial production, thereby contributing to improved surface quality of continuously cast slabs.

## 1. Introduction

Railway gondola cars constitute the most numerous and widely used type of railway freight car and are primarily responsible for transporting bulk materials such as coal, ore, and construction materials; therefore, their operational safety and reliability are of critical importance. Due to significant regional variations in climatic conditions, particularly in southern coastal areas, the protective coating inside the car body is rapidly deteriorated under high-salinity and high-humidity service environments combined with impact and friction from transported goods, thereby resulting in accelerated corrosion of the car body [1,2,3]. Since coal and ore typically contain sulfide compounds, an acidic corrosive environment is likely to form at the bottom of gondola cars under humid atmospheric conditions, thereby imposing more stringent performance requirements on the steels used for their manufacture. While traditional weathering steels based on the Cr–Ni–Cu compositional system exhibit favorable durability under atmospheric corrosion conditions, they exhibit limited resistance to prolonged exposure in highly acidic localized environments. Consequently, their corrosion resistance in acidic environments requires further improvement [4,5].

In recent years, studies have shown that the addition of antimony (Sb) can significantly enhance the acid corrosion resistance of steel. Sb significantly improves the acid corrosion resistance of low-alloy steels by promoting the formation of a protective oxide film enriched with Sb_2_O_3_ and Sb_3_O_5_ on the steel surface and by suppressing electrochemical corrosion reactions [6,7,8,9,10]. These findings provide an important theoretical foundation for the development of a new generation of Sb-containing high-corrosion-resistant steels and promote the engineering application of such materials [11,12].

In industrial production, such low-alloy corrosion-resistant steels are typically manufactured through continuous casting and rolling, which are characterized by process simplicity and low cost. Therefore, the internal quality of continuously cast slabs directly determines the performance and service reliability of the final product, and hot ductility is a key property used to evaluate slab quality. During the straightening stage of the continuous casting process, insufficient hot ductility can readily lead to the formation of defects, such as transverse or corner cracks, on the slab surface. These defects not only significantly reduce yield and surface quality but also adversely affect subsequent production efficiency and service safety [13,14]. Extensive studies have been conducted on continuous casting cracks, and it has been found that low-alloy steels typically exhibit a pronounced ductility trough within the straightening temperature range of 650~1000 °C [15,16]. Previous studies [17,18,19] have indicated that the reduction in hot ductility within this temperature interval is associated with the precipitation of film-like proeutectoid ferrite along austenite grain boundaries. Because ferrite exhibits lower strength than austenite, severe stress concentration tends to occur at the grain-boundary ferrite film, thereby promoting premature crack initiation and eventual fracture. Furthermore, in microalloyed steels, carbides and nitrides of Nb, V, and Ti precipitate as second-phase particles along grain boundaries. Under tensile stress, these second-phase particles readily act as crack initiation sites, leading to intergranular fracture and a significant deterioration in hot ductility [20,21,22]. In addition to grain-boundary ferrite and second-phase precipitates, the segregation of impurity elements (e.g., S, P, Cu, Sn, and Sb) at ferrite/austenite interfaces and austenite grain boundaries constitutes another important factor influencing hot ductility. Nachtrab et al. [23] investigated the effects of Sn, Sb, and Cu on the hot ductility of C–Mn steels. The results indicated that these elements were significantly enriched along austenite grain boundaries under high-temperature deformation conditions, thereby severely deteriorating hot ductility. Harada et al. [24] demonstrated that the formation of transverse cracks on the surface of continuously cast slabs was closely associated with the localized segregation of P at austenite grain boundaries.

Although the aforementioned studies have demonstrated the detrimental effect of Sb on the hot ductility of low-alloy corrosion-resistant steel, the specific effect of Sb concentration on hot ductility has not yet been fully clarified. Sun et al. [25] reported that the addition of 0.16 wt% Sb to C–Mn steel resulted in Sb segregation at grain boundaries, thereby broadening the hot ductility trough and decreasing the reduction in area within the temperature range of 750–950 °C. However, He et al. [26] observed that the addition of 0.05 wt% Sb to microalloyed steel did not result in grain boundary segregation and exerted no significant effect on hot ductility. Therefore, the Sb content plays a critical role in determining the hot ductility of steel, and its detrimental effect becomes apparent only when the Sb concentration exceeds a certain threshold. Since the influence of Sb concentration on the hot ductility curve varies depending on steel composition, optimization of the continuous casting process requires identification of the corresponding ductility trough. Hence, a systematic investigation into the effects of Sb concentration on hot ductility evolution and fracture behavior in low-alloy corrosion-resistant steel is urgently required.

In this study, the effect of Sb on the hot ductility and fracture behavior of steel at elevated temperatures was systematically investigated. The temperature range of the ductility trough and its formation mechanism were clarified. These findings can provide a theoretical basis and technical reference for the continuous casting process parameters in industrial production, thereby contributing to the prevention of surface cracking in continuous casting slabs.

## 2. Materials and Methods

**Figure 1:**
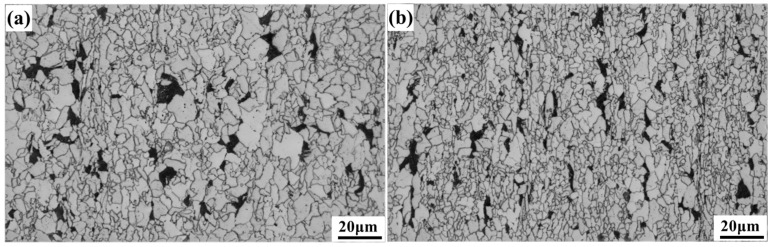
Microstructures of (**a**) 0.09Sb steel and (**b**) 0.15Sb steel.

Two low-alloy corrosion-resistant steels with different Sb contents were prepared using a vacuum induction melting furnace and were designated as 0.09Sb and 0.15Sb steels, respectively. The detailed chemical compositions are presented in Table 1. Figure 1 illustrates the initial microstructures of the two experimental steels. Both the 0.09Sb steel and the 0.15Sb steel are composed of ferrite and pearlite; however, the ferrite grains in the 0.15Sb steel are noticeably finer than those in the 0.09Sb steel. The specimens used for high-temperature tensile testing were machined directly from the as-cast ingots after smelting, with a diameter of 10 mm, a length of 80 mm, and an effective uniform heating zone of approximately 20 mm.

**Figure 2:**
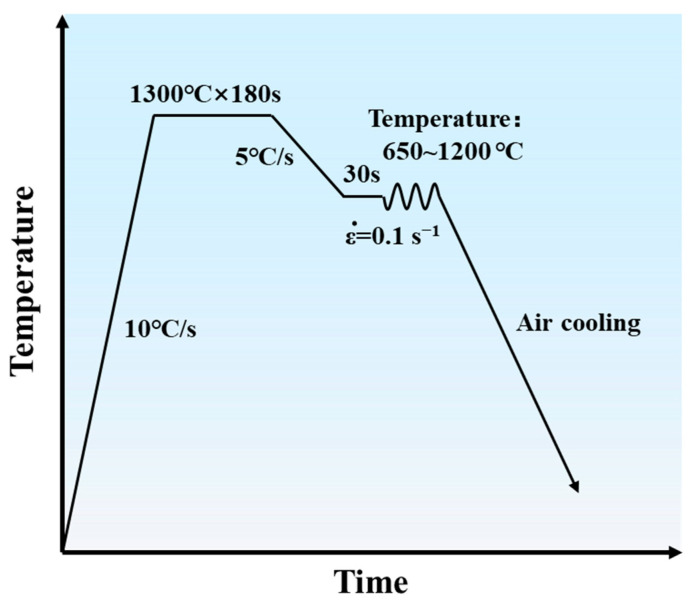
Schematic diagram of the hot ductility test procedure.

The hot ductility of 0.09Sb and 0.15Sb steels over the temperature range of 650–1200 °C was evaluated using an in-house developed MMS-200 thermal-mechanical simulation at Northeastern University, China. A protective atmosphere was employed during testing to prevent oxidation. A schematic illustration of the high-temperature tensile testing procedure is presented in Figure 2.

**Figure 3:**
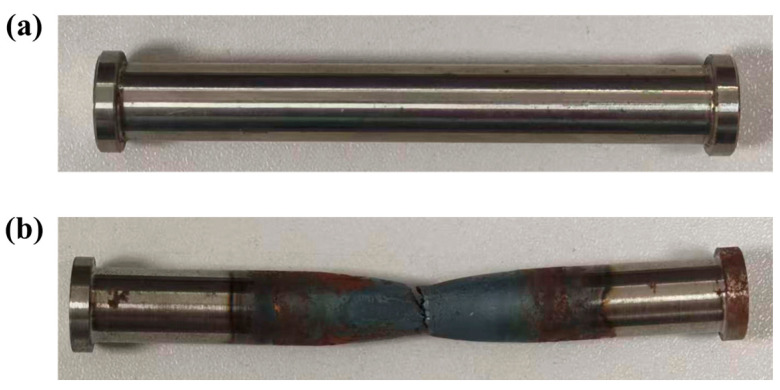
Image of the specimen (**a**) before the high-temperature tensile test and (**b**) after fracture.

The specimens were rapidly heated to 1300 °C and held for 180 s to ensure the dissolution of precipitates and the complete solid solution of alloying elements. Subsequently, the specimens were cooled to the deformation temperatures (1200, 1050, 900, 850, 800, 750, 700, and 650 °C) at a rate of 5 °C/s. After holding for 30 s at the target temperature, the specimens were subjected to tensile deformation until fracture at a strain rate of 0.1 s^−1^. The strain rate of 0.1 s^−1^ was selected based on reference to the continuous-casting and straightening process parameters commonly used in the industrial production of low-alloy corrosion-resistant steels. Immediately after fracture, the specimens were quenched using high-speed nitrogen gas to rapidly cool them to room temperature, thereby preserving the microstructural characteristics corresponding to each deformation temperature. The morphologies of the samples before and after fracture the high-temperature tensile tests are shown in Figure 3.

Prior to high-temperature tensile testing, the initial diameter of each specimen was measured and recorded using a Vernier caliper. After fracture, the minimum diameter at the necked region was measured. The reduction in area (RA) at different deformation temperatures was calculated according to Equation (1).

(1) $$RA=\frac{A_{0}-A_{1}}{A_{0}}\times 100\%$$

where *RA* is the reduction in area of the specimen (%); *A*_0_ is the initial cross-sectional area (mm^2^); and *A*_1_ is the minimum cross-sectional area at the necked region after fracture (mm^2^).

The longitudinal sections of the fractured specimens were prepared using electrical discharge machining and subsequently processed into metallographic specimens. After grinding with 400–1500 grit SiC papers and mechanical polishing, the specimens were etched using a saturated picric acid solution. The microstructural characteristics near the fracture surfaces were examined using the OLYMPUS optical microscopy (OM, Tokyo, Japan) and the JEOL-8530 F electron probe microanalysis (EPMA, Tokyo, Japan). The fracture morphologies were examined using the FEI QUANTA 600 scanning electron microscopy (SEM, Hillsboro, OR, USA). The equilibrium precipitation phase diagrams of the tested steels were calculated using Thermo-Calc software (TCFE9 database, 2023a), and the relationship between phase composition and temperature was analyzed.

## 3. Results

### 3.1. Hot Ductility

The RA was used to evaluate the hot ductility of the tested steels at elevated temperatures. Generally, when RA exceeds 60%, the casting slab exhibits good hot ductility and can withstand external deformation without the formation of surface cracks; when RA falls below 60%, the susceptibility of the casting slab to crack formation increases and becomes more pronounced with further decreases in RA [27,28]. Therefore, in this study, the temperature corresponding to an RA of 60% was adopted as the criterion for distinguishing between the ductile and brittle regions, and the temperature range within which RA is less than 60% was defined as the ductility trough.

**Figure 4:**
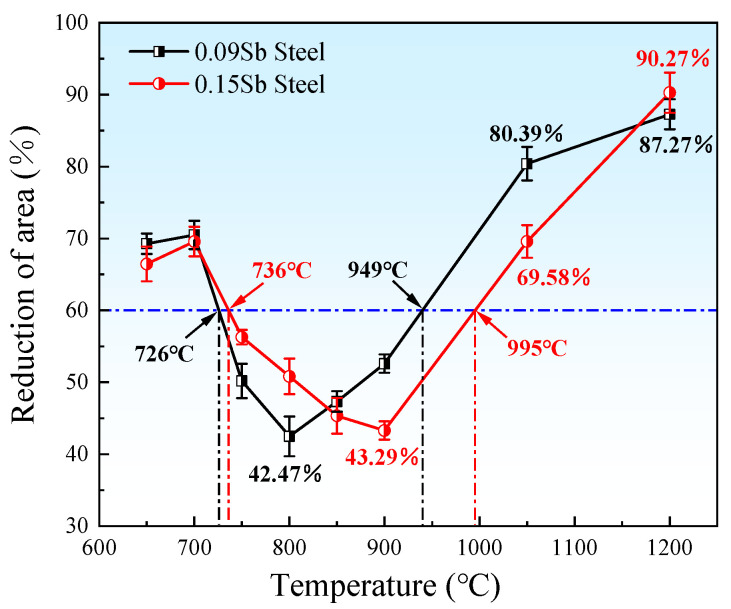
Hot ductility curves of experimental steels.

Figure 4 presents the hot ductility curves of the two experimental steels over the temperature range of 650–1200 °C. Under tensile deformation, both steels exhibit a typical V-shaped curve with a distinct ductility trough. However, the temperature ranges of the ductility troughs differ between the two steels. Specifically, the ductility trough of the 0.09Sb steel spans 726–949 °C, whereas that of the 0.15Sb steel spans 736–995 °C. The minimum hot ductility of the 0.09Sb steel occurs at 800 °C, corresponding to the RA of 42.47%. For the 0.15Sb steel, the minimum hot ductility occurs at 900 °C, with the RA of 43.29%. Both steels achieve maximum hot ductility at 1200 °C, with RA values of 87.27% and 90.27% for the 0.09Sb and 0.15Sb steels, respectively. Consequently, increasing the Sb content broadens the ductility trough temperature range and shifts the temperature corresponding to the minimum ductility toward higher values.

### 3.2. Fracture Morphology

**Figure 5:**
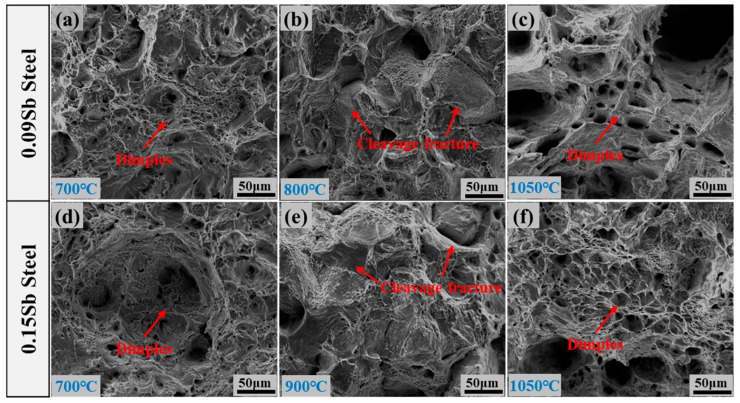
Fracture morphologies of specimens tested at different temperatures. (**a**) 0.09Sb Steel-700 °C; (**b**) 0.09Sb Steel-800 °C; (**c**) 0.09Sb Steel-1050 °C; (**d**) 0.15Sb Steel-700 °C; (**e**) 0.15Sb Steel-900 °C; (**f**) 0.15Sb Steel-1050 °C.

The fracture mechanisms of the experimental steels at different deformation temperatures were further analyzed based on fracture morphology observations, as shown in Figure 5. Figure 5a,d show the fracture morphologies of the 0.09Sb and 0.15Sb steels at 700 °C, respectively. Both exhibit a dimpled morphology characteristic of typical ductile fracture, indicating good hot ductility.

Figure 5b,e present the fracture morphologies of the 0.09Sb steel at 800 °C and the 0.15Sb steel at 900 °C, respectively. At these temperatures, the number of dimples is significantly reduced, and the fractures exhibit a sugar-like appearance characterized by smooth and flat facets outlining the prior austenite grain boundaries. This morphology indicates a typical intergranular brittle fracture mechanism in both steels. Intergranular brittle fracture occurs because the load-bearing capacity and bonding strength of the grain boundaries are lower than those of the grain interiors. Under tensile loading, cracks preferentially propagate along these weakened grain boundaries, leading to intergranular failure. Grain boundary weakening generally arises from two primary mechanisms: (i) the segregation of impurity elements or solute atoms at grain boundaries, such as the enrichment of trace elements (e.g., S, Sn, and Sb), which markedly reduces grain boundary cohesive energy [23,29]; and (ii) the precipitation of second-phase particles at grain boundaries, where hard and brittle particles induce stress concentration under tensile loading, serving as crack initiation sites and thereby degrading grain boundary strength [21,30]. Consequently, the hot ductility of both the 0.09Sb and 0.15Sb steels was significantly reduced, with the RA reaching its minimum value. When the deformation temperature increased to 1050 °C (Figure 5c,f), the fracture morphologies of both steels reverted to a dimpled morphology, indicating improved hot ductility at this temperature, which corresponded to the maximum RA. In summary, fracture morphology is closely correlated with hot ductility.

### 3.3. Microstructure near Fracture

**Figure 6:**
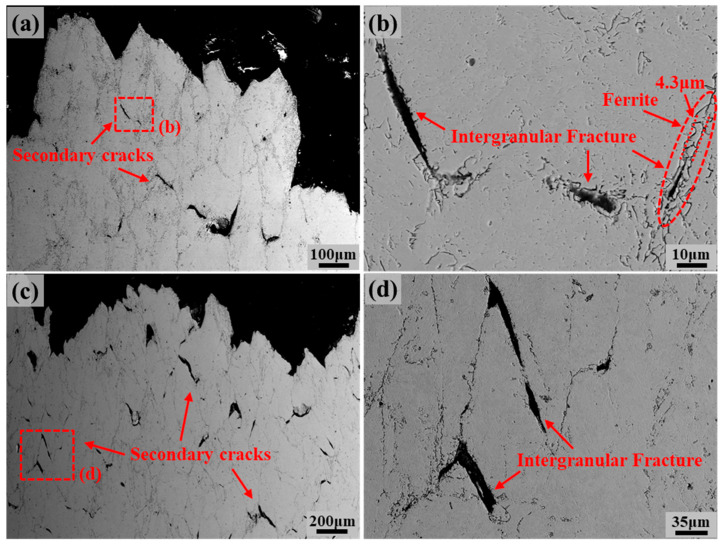
Microstructure near the fracture surface of the experimental steels within the ductility trough. (**a**) Fracture surface microstructure of the 0.09Sb steel deformed at 800 °C. (**b**) Higher magnification image of the marked area in (**a**), showing intergranular fracture along the intergranular ferrite. (**c**) Fracture surface microstructure of the 0.15Sb steel deformed at 900 °C. (**d**) Higher magnification image of the marked area in (**c**), showing characteristics of intergranular fracture.

To elucidate the crack initiation and propagation mechanisms of the experimental steels within the ductility trough, the longitudinal microstructures adjacent to the fracture surfaces were characterized. After etching in a saturated aqueous picric acid solution, the prior austenite microstructure and grain boundaries were clearly revealed. Figure 6a shows the microstructure near the fracture surface of the 0.09Sb steel deformed at 800 °C, where several intergranular secondary cracks are observed adjacent to the primary fracture. A higher-magnification image (Figure 6b) reveals a ferrite film approximately 4.3 μm thick formed along the prior austenite grain boundaries. Cracks originate within this ferrite layer and propagate along the grain boundaries, indicating a strong correlation between intergranular fracture and grain boundary ferrite. Figure 6c shows the microstructure near the fracture surface of the 0.15Sb steel deformed at 900 °C. Numerous intergranular secondary cracks are also present; however, no evident grain boundary ferrite is detected along the prior austenite grain boundaries in this case.

**Figure 7:**
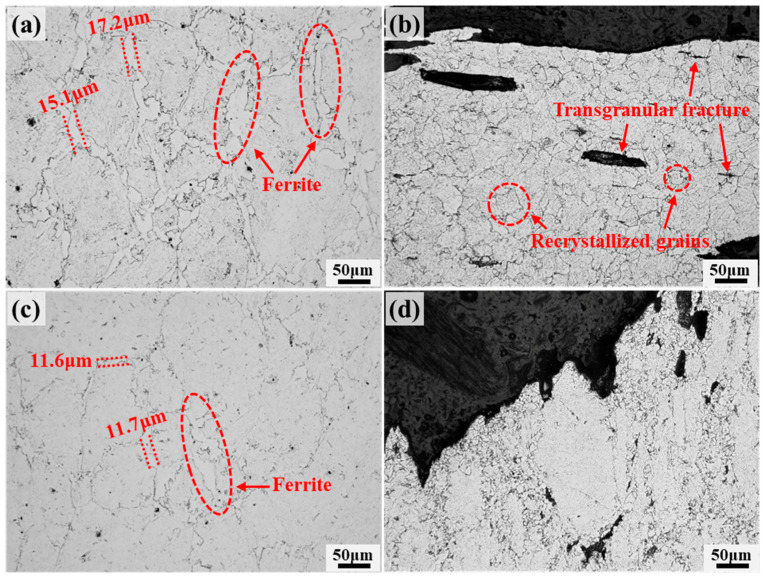
Microstructure near the fracture surface of the tested steels in the ductile region. (**a**) 0.09Sb Steel-700 °C (**b**) 0.09Sb Steel-1050 °C; (**c**) 0.15Sb Steel-700 °C; (**d**) 0.15Sb Steel-1050 °C.

Figure 7 presents the microstructures near the fracture surfaces of the 0.09Sb and 0.15Sb steels deformed at 700 °C and 1050 °C. As shown in Figure 7a,c, when the deformation temperature is 700 °C, a relatively thick ferrite layer is clearly observed along the grain boundaries in both steels, and no intergranular secondary cracks are detected. The grain boundary ferrite is thicker in the 0.09Sb steel, measuring approximately 15–17 μm, whereas that in the 0.15Sb steel is about 11 μm. These results indicate that the thickness of the grain boundary ferrite exerts a significant influence on hot ductility. When deformed at 1050 °C, as shown in Figure 7b, the fracture microstructure of the 0.09Sb steel consists of uniform and fine recrystallized grains, with cracks propagating in a transgranular manner and exhibiting typical ductile fracture characteristics. Figure 7d shows the microstructure near the fracture surface of the 0.15Sb steel at 1050 °C, where several microvoids are observed, along with small and indistinct newly formed grains along the prior austenite grain boundaries, indicating that the steel remains in the grain boundary migration stage of dynamic recrystallization. Driven by the stored deformation energy, grain boundaries migrate toward regions of higher dislocation density and continue to grow, forming equiaxed recrystallized grains. In contrast, dynamic recrystallization in the 0.09Sb steel is essentially complete, whereas the 0.15Sb steel remains at the initial stage. These results suggest that increasing Sb content elevates the dynamic recrystallization temperature and suppresses recrystallization kinetics in the tested steel. Consequently, at 1050 °C, the hot ductility of the 0.09Sb steel is significantly superior to that of the 0.15Sb steel.

### 3.4. True Stress–True Strain Curves

**Figure 8:**
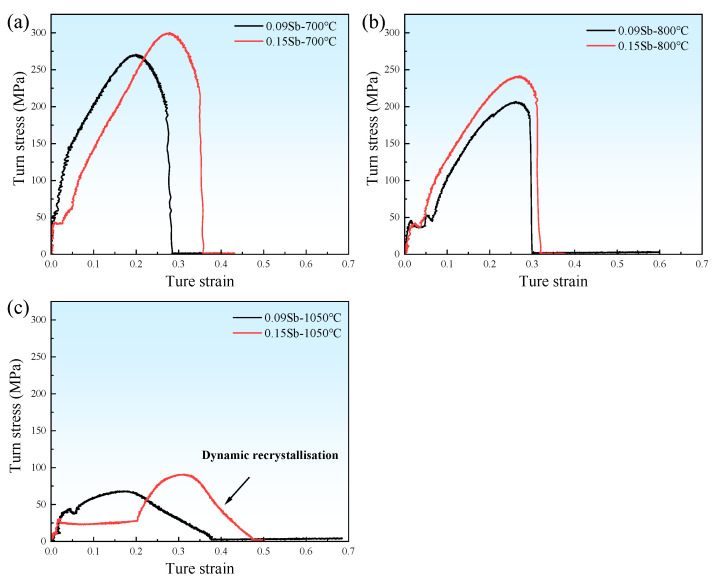
True stress–true strain curves of the experimental steels at different deformation temperatures. (**a**) 700 °C; (**b**) 800 °C; (**c**) 1050 °C.

To investigate whether dynamic recrystallisation occurred during high-temperature tensile testing, true stress–true strain curves were plotted for the two experimental steels at 700 °C, 800 °C, and 1050 °C, as shown in Figure 8. As shown in Figure 8a, at 700 °C, the true stress of both experimental steels decreased sharply after reaching the peak stress until fracture occurred, indicating that no significant dynamic recrystallisation occurred during deformation. When the temperature was increased to 800 °C, as shown in Figure 8b, the true stress of both experimental steels similarly decreased rapidly after reaching the peak stress, indicating that dynamic recrystallisation still did not occur at this temperature. However, when the temperature was further increased to 1050 °C, as shown in Figure 8c, the true stress of both experimental steels decreased gradually after reaching the peak stress, indicating that extensive dynamic recrystallisation occurred during hot deformation. As the deformation temperature increases, the peak stress of both experimental steels gradually decreases. It is worth noting that, at different deformation temperatures, the peak stress of the 0.15Sb steel is consistently higher than that of the 0.09Sb steel, indicating that the 0.15Sb steel possesses higher high-temperature strength.

## 4. Discussion

### 4.1. The Effect of Ferrite Transformation on Hot Ductility

**Figure 9:**
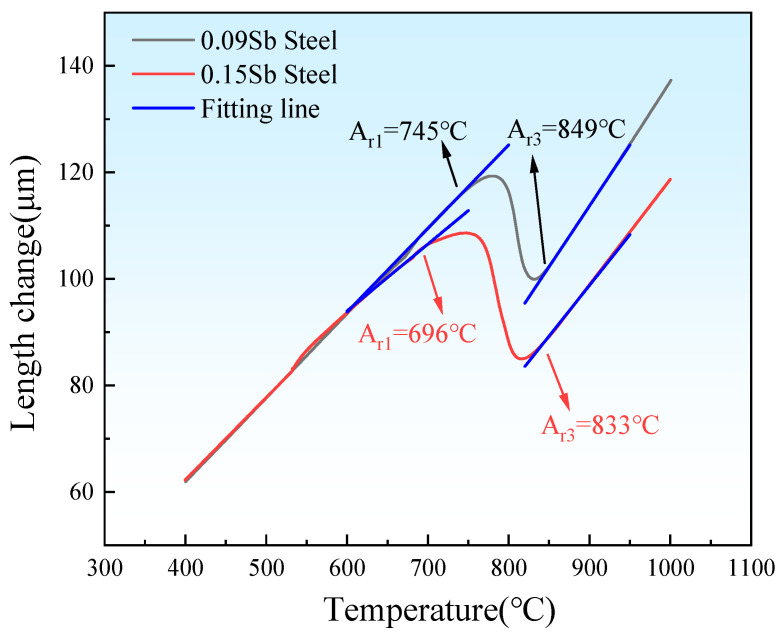
Temperature curves for the austenite-to-ferrite transformation of the experimental steels.

Based on Figure 6 and Figure 7, the influence of grain boundary ferrite on hot ductility is primarily governed by its thickness and distribution morphology. Accordingly, the austenite-to-ferrite transformation temperature curves of the two tested steels were determined. The transformation start and finish temperatures were determined using the tangent method (Figure 9). The transformation temperature ranges of the 0.09Sb and 0.15Sb steels are 745–849 °C and 696–833 °C, respectively. With increasing Sb content, the austenite-to-ferrite transformation start temperature decreases from 849 °C to 833 °C, while the transformation temperature range expands from 104 °C to 137 °C. These results indicate that Sb content significantly affects the phase transformation behavior of the steel. It can therefore be concluded that Sb content has a significant effect on the phase transformation behavior of steel. Sb preferentially segregates at grain boundaries, thereby reducing the grain boundary interfacial energy. Since ferrite preferentially nucleates at high-energy grain boundaries, the reduction in interfacial energy suppresses ferrite nucleation and requires a greater degree of undercooling to initiate the phase transformation, thereby decreasing the austenite-to-ferrite transformation start temperature (A_r3)_. Furthermore, during ferrite growth, Sb atoms segregate at the moving austenite/ferrite interface, generating a “solute drag” effect. This hinders interface migration and delays completion of the phase transformation, thereby lowering the austenite-to-ferrite transformation finish temperature (A_r1_).

During isothermal holding at 800 °C, ferrite preferentially nucleates at prior austenite grain boundaries and grows laterally along them. This process promotes the formation of continuous or semi-continuous ferrite films along the grain boundaries. Owing to the significant strength difference between ferrite and austenite, with the deformation strength of ferrite being approximately one-quarter that of austenite [17,26], ferrite undergoes preferential plastic deformation during high-temperature loading. However, because the ferrite layer formed at this stage is very thin (approximately 4.3 μm) and exhibits a network morphology, it cannot effectively accommodate or relax applied stress, resulting in severe stress concentration. Once microcracks initiate, they rapidly propagate along the grain boundary ferrite film, ultimately leading to intergranular fracture. This mechanism accounts for the intergranular brittle fracture observed in the 0.09Sb steel at 800 °C. When the deformation temperature was reduced to 700 °C, the increased undercooling enhanced the driving force for phase transformation, resulting in a greater extent of austenite-to-ferrite transformation and increasing the thickness of the ferrite layer to 11–17 μm. The thicker grain-boundary ferrite layer provided sufficient volume to accommodate the strain incompatibility between the two phases, thereby reducing stress concentration at the grain boundaries through plastic deformation and minimizing microcrack initiation. Furthermore, when microcracks initiated at the grain boundaries and propagated into the ferrite layer, the increased ferrite thickness effectively blunted the crack tips, thereby increasing resistance to crack propagation and enhancing energy dissipation. Consequently, the fracture mode transitioned from complete intergranular brittle fracture to a dimpled ductile morphology, and both the 0.09Sb and 0.15Sb steels exhibited improved hot ductility at 700 °C.

### 4.2. The Effect of Precipitates on Hot Ductility

**Figure 10:**
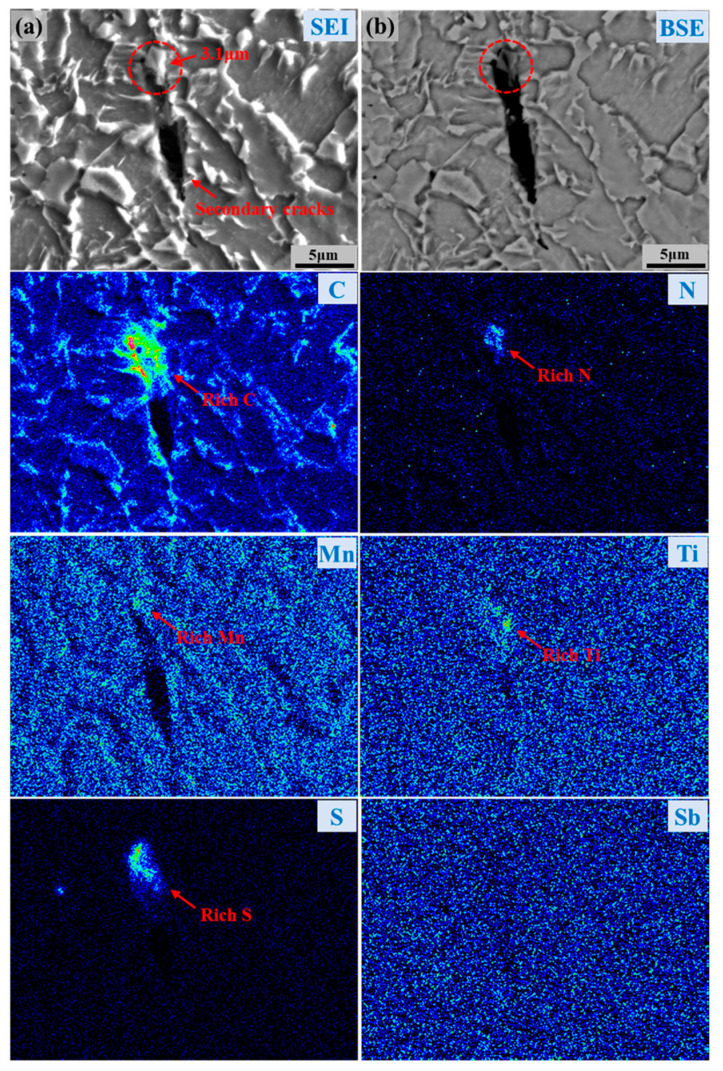
Elemental analysis of precipitates in the fracture microstructure of the 0.15Sb steel deformed at 900 °C. (**a**) Secondary electron image; (**b**) Backscattered electron image.

To further elucidate the reduced hot ductility of the 0.15Sb steel at 900 °C, where intergranular brittle fracture was observed in the absence of significant grain-boundary ferrite formation (Figure 6c,d), elemental mapping analysis was performed on the fracture surface regions containing secondary cracks. The results are shown in Figure 10. Cracks were observed to originate from second-phase particles enriched in C, Mn, Ti, N, and S, with an average size of approximately 3.1 μm. Therefore, large composite second-phase particles consisting of Ti(C,N) and MnS are inferred to be a primary factor contributing to the brittle fracture at 900 °C, and processing within this temperature range should be avoided. Under high-temperature deformation, plastic strain localizes in the relatively soft austenite matrix, whereas the hard second-phase particles resist deformation, resulting in stress concentration at the particle–matrix interface [22,26,28]. As the applied stress increases beyond the interfacial bonding strength, particle–matrix debonding occurs, leading to microvoid formation. With continued deformation, microvoids nucleated at second-phase particles grow and coalesce into cracks, which propagate rapidly and ultimately lead to macroscopic fracture. The energy consumed during this process is significantly lower than that required for matrix tearing, thereby substantially reducing hot ductility. Notably, elemental mapping of the precipitates in Figure 10 did not reveal significant Sb segregation, indicating that Sb does not exist in the steel in the form of discrete precipitates.

**Figure 11:**
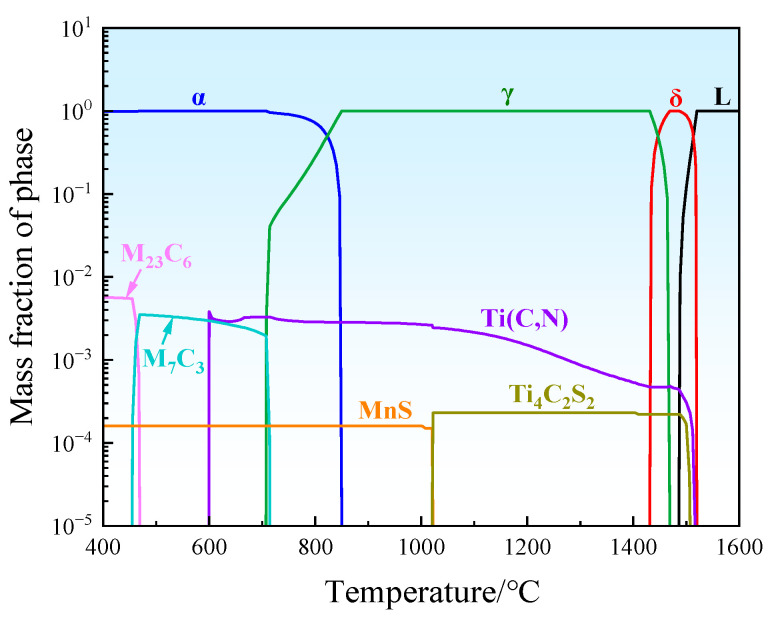
Precipitation phase diagram of the 0.15Sb steel as a function of temperature calculated by Thermo-Calc software.

At high temperatures, alloying elements exist in solid solution in the steel matrix. As the temperature decreases, their solid solubility gradually diminishes, leading to the formation of second-phase particles with compositions and crystal structures distinct from those of the matrix, which ultimately precipitate as discrete phases. To investigate the temperature-dependent precipitation behavior, the equilibrium precipitate phase diagram of the 0.15Sb steel was calculated using Thermo-Calc software. As shown in Figure 11, the primary precipitates include Ti(C,N), Ti_4_C_2_S_2_, MnS, M_7_C_3_, and M_23_C_6_, where the M_7_C_3_- and M_23_C_6_-type precipitates are mainly (Cr,Fe)_7_C_3_ and (Cr,Fe,Mn)_23_C_6_, respectively. With decreasing temperature, second-phase particles progressively precipitate. The Ti-bearing precipitates are predominantly Ti_4_C_2_S_2_ and Ti(C,N). Ti_4_C_2_S_2_ primarily precipitates at temperatures above 1020 °C, whereas within the ductility trough, Ti preferentially precipitates in the form of Ti(C,N). In addition, MnS precipitation becomes significant below approximately 1020 °C. Therefore, the dominant precipitates within the ductility trough are Ti(C,N) and MnS. When large second-phase particles precipitate at grain boundaries, the grain-boundary cohesive strength is significantly reduced, resulting in stress concentration and microvoid formation. Under applied stress, these microvoids grow and coalesce with adjacent voids, forming cracks that propagate rapidly along grain boundaries and ultimately lead to brittle fracture.

### 4.3. The Effect of Non-Equilibrium Grain Boundary Segregation of Sb on Hot Ductility

Previous studies [25,31] have indicated that, in addition to grain-boundary ferrite films and large-sized precipitates, Sb segregation at grain boundaries significantly reduces grain-boundary strength and deteriorates hot ductility. This segregation phenomenon can be categorized into two types: equilibrium and non-equilibrium grain-boundary segregation. Equilibrium grain-boundary segregation is governed by thermodynamic equilibrium, with the reduction in interfacial free energy serving as its driving force. The enrichment zone at the grain boundary is extremely narrow, typically only a few atomic spacings, and at a given temperature, the degree of equilibrium segregation reaches a stable value [32]. In contrast, non-equilibrium grain-boundary segregation is a kinetic process primarily driven by the diffusion of solute–point defect complexes toward grain boundaries. It is commonly observed during rapid quenching, high-energy particle irradiation, low-stress elastic deformation, and high-temperature plastic deformation [33,34,35]. In this study, when the specimens were rapidly cooled from 1300 °C, vacancies at the grain boundaries were rapidly annihilated. However, supersaturated Sb–vacancy complexes remained within the grain interior. This condition generated a concentration gradient of these complexes between the grain interior and the grain boundaries, providing the driving force for their diffusion toward the grain boundaries and ultimately resulting in non-equilibrium grain-boundary segregation of Sb.

In 1957, McLean [32] proposed a thermodynamic model describing equilibrium grain-boundary segregation. At a constant temperature, the maximum equilibrium grain-boundary segregation concentration of the solute, *C*_eq_, can be expressed as:

(2) $$C_{\mathrm{eq}}=\frac{C_{g}\exp(E_{\mathrm{gb}}/kT)}{1+C_{g}\exp(E_{\mathrm{gb}}/kT)}$$

where *C*_g_ denotes the solute concentration in the matrix (wt.%); *E*_gb_ is the grain-boundary segregation free energy (eV); *k* is the Boltzmann constant (eV/K); and *T* is the absolute temperature (K). According to Equation (2), the equilibrium segregation concentration decreases with increasing temperature, indicating that within the hot-ductility temperature range, the equilibrium segregation level remains relatively low.

Studies [35,36] have shown that when specimens are rapidly cooled from a higher temperature to a lower temperature and subsequently isothermally held, the extent of non-equilibrium grain-boundary segregation initially increases with holding time, reaches a maximum at a critical time, and then decreases with prolonged holding, ultimately resulting in desegregation or even complete disappearance of segregation. Therefore, to evaluate this maximum segregation level, the isothermal kinetic model for non-equilibrium grain-boundary segregation was adopted [37]. When the specimen is cooled from the solution treatment temperature *T*_i_ to the deformation temperature *T*_i+1_, the maximum grain-boundary segregation concentration of the solute, *C*_b_(*T*_i+1_), can be calculated according to Equation (3).

(3) $$C_{b}(T_{i+1})=C_{g}(\frac{E_{b}}{E_{f}})\exp(\frac{E_{b}-E_{f}}{kT_{i}}-\frac{E_{b}-E_{f}}{kT_{i+1}})$$

where *E*_b_ denotes the binding energy between the solute atom and a vacancy (eV), and *E*_f_ represents the vacancy formation energy of the matrix (eV). It can be derived that *C*_b_(*T*_i+1_) depends solely on the solution treatment temperature *T*_i_ and the deformation temperature *T*_i+1_, and is independent of the cooling rate.

During isothermal holding, attainment of the maximum grain-boundary segregation concentration requires a kinetic process. Therefore, a critical time, *t*_c_, is defined. At this critical time, the diffusion flux of solute–vacancy complexes from the grain interior to the grain boundary is balanced by the counter-diffusion flux of solute atoms from the grain boundary to the grain interior, at which point the segregation concentration attains its maximum value. The critical time *t*_c_ can be calculated according to Equation (4).

(4) $$t_{c}=\frac{\beta^{2}\ln(D_{c}/D_{s})}{4\delta (D_{c}-D_{s})}$$

where β denotes the grain radius (μm); *D*_c_ is the diffusion coefficient of the solute–vacancy complex (m^2^·s^−1^); *D*_s_ is the diffusion coefficient of the solute (m^2^·s^−1^); and *δ* is the critical time constant.

When the isothermal holding time is shorter than the critical time *t*_c_, the process is predominantly governed by the segregation of Sb atom–vacancy complexes from the grain interior to the grain boundaries. The kinetic equation describing this process is given by Equation (5).

(5) $$\frac{C_{b}(t)-C_{g}}{C_{m}(T_{i+1})-C_{g}}=1-\exp(\frac{4D_{c}t}{{\alpha_{i+1}}^{2}d^{2}})\mathrm{erfc}(\frac{\sqrt{4D_{c}t}}{\alpha_{i+1}d})$$

where *C*_b_(*t*) represents the grain boundary segregation concentration at the isothermal holding time *t* (wt.%); *C*_m_(*T*_i+1_) denotes the maximum grain boundary segregation concentration at temperature *T*_i+1_ (wt.%); *d* is the segregation layer thickness (μm); and *α*_i+1_ is the maximum enrichment ratio, expressed as *α*_i+1_ = *C*_m_(*T*_i+1_)/*C*_g_.

When the isothermal holding time exceeds the critical time *t*_c_, the process is predominantly governed by the desegregation of Sb atoms from the grain boundaries into the grain interior. The kinetic equation describing this process is given by Equation (6).

(6) $$C_{b}(t)=C_{g}+\frac{1}{2}\left[C_{b}(t_{c})-C_{g}\right]\times \left\{\mathrm{erf}\left[\frac{d/2}{\sqrt{4D_{s}(t-t_{c})}}\right]-\mathrm{erf}\left[-\frac{d/2}{\sqrt{4D_{s}(t-t_{c})}}\right]\right\}$$

**Figure 12:**
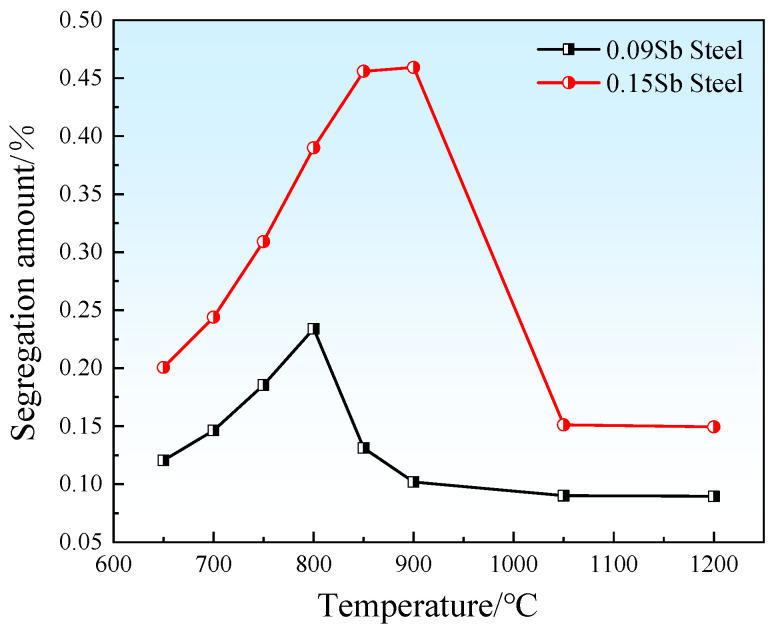
Predicted non-equilibrium grain boundary segregation concentrations of the experimental steels during high-temperature tensile testing.

The aforementioned model is applicable under the conditions of the present study and can be employed to predict the grain boundary segregation behavior of Sb during high-temperature tensile testing. Accordingly, calculations were performed for the 0.09Sb and 0.15Sb steels, and the results are presented in Figure 12. The parameters used in the calculations are summarized in Table 2. It is evident that the 0.09Sb and 0.15Sb steels reach peak Sb segregation at 800 °C and 900 °C, respectively. These temperatures correspond to the ductility troughs observed in Figure 4, thereby demonstrating the strong predictive capability of the model. Sb segregation displaces matrix metal atoms at the grain boundaries, thereby disrupting metallic bonding and significantly reducing the atomic bonding strength within the grain boundary region. Consequently, during high-temperature plastic deformation, stress preferentially concentrates at these weakened grain boundaries. Because the grain boundaries are unable to undergo coordinated sliding, intergranular cracking readily occurs [38]. As a result, the reduction in area decreases sharply, leading to a marked deterioration in hot ductility.

As shown in Figure 12, the peak segregation concentration of the 0.15Sb steel reaches 0.46 wt%, which is higher than that of the 0.09Sb steel (0.23 wt%). This indicates that non-equilibrium grain boundary segregation of Sb is a key factor responsible for broadening the hot ductility trough and shifting it toward higher temperatures. The non-equilibrium grain boundary segregation concentration of Sb exhibits a trend of initially increasing and subsequently decreasing with decreasing temperature. This behavior can be attributed to the relatively rapid solute diffusion rate at high temperatures, resulting in a short critical segregation time. Once the isothermal holding time exceeds the critical time, desegregation occurs, leading to a relatively low level of non-equilibrium grain boundary segregation. At lower temperatures, diffusion of solute–vacancy complexes is significantly hindered, thereby impeding attainment of the maximum non-equilibrium grain boundary segregation level. Therefore, the peak non-equilibrium grain boundary segregation of Sb is achieved only when thermodynamic and kinetic conditions are optimally balanced [36].

**Figure 13:**
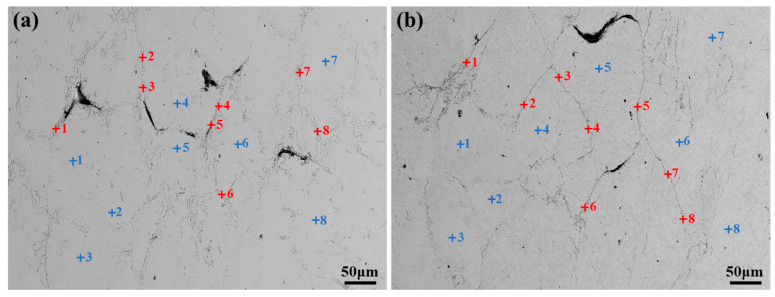
The Sb concentrations at the grain boundaries and within the grains of the experimental steel were determined by EPMA point-scanning analysis. (**a**) 0.09Sb Steel-800 °C; (**b**) 0.15Sb Steel-900 °C.

**Figure 14:**
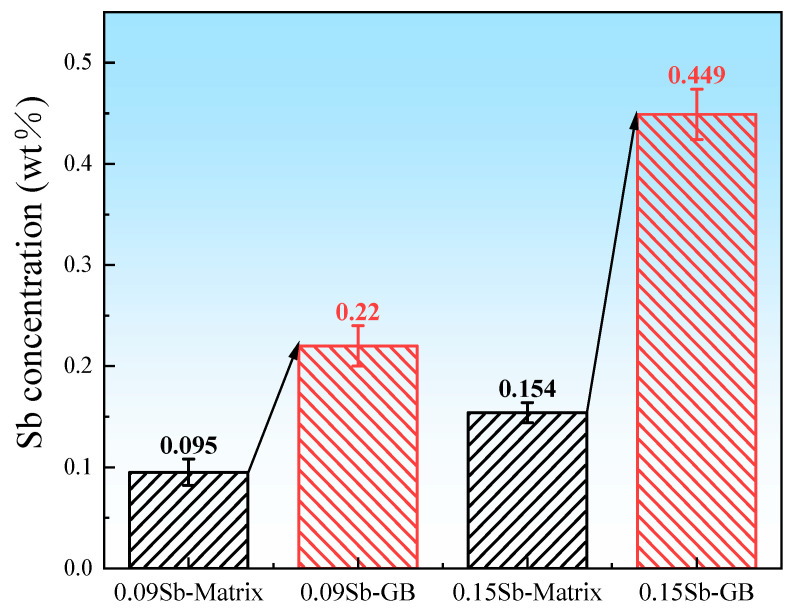
Sb concentrations measured at the grain boundaries and within the grains of the experimental steel.

To further investigate the effect of grain boundary Sb segregation on hot ductility, quantitative elemental analyses were conducted on the 0.09Sb–800 °C and 0.15Sb–900 °C samples using EPMA point-scanning techniques. As shown in Figure 13, quantitative Sb measurements were obtained from eight randomly selected locations at the grain boundaries and within the grains, respectively. The results are summarized in Table 3 and Figure 14. The red and blue markers in Figure 13 represent the EPMA point-scanning locations for Sb at the grain boundaries and within the grains, respectively. Both experimental steels were found to exhibit Sb grain boundary segregation. In the 0.09Sb steel, the Sb concentration at the grain boundaries at 800 °C reached 0.22 wt%, which was approximately 2.3 times higher than that in the matrix; in the 0.15Sb steel, the Sb concentration at the grain boundaries at 900 °C reached 0.449 wt%, approximately 2.9 times higher than that in the matrix. It can therefore be concluded that an increase in Sb content leads to enhanced Sb segregation at the grain boundaries. Furthermore, a comparison of Figure 12 and Figure 14 reveals that the Sb grain boundary segregation values predicted by the model are in good agreement with the experimental results, indicating that the established non-equilibrium Sb grain boundary segregation model is applicable to the present study.

### 4.4. Fracture Mechanism in the Ductility Trough

**Figure 15:**
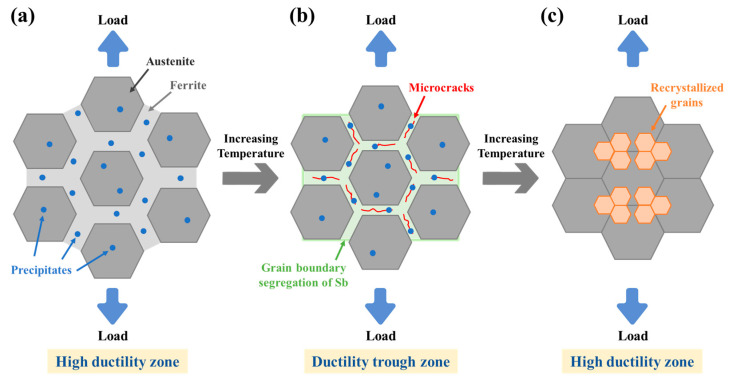
Schematic illustration of the fracture mechanism of the experimental steels within the ductility trough. (**a**) The low-temperature high-ductility zone; (**b**) the ductility trough zone; (**c**) the high-temperature high-ductility zone.

Figure 15 presents a schematic illustration of the fracture mechanism of the experimental steels within the ductility trough. Within the temperature range of 650–1200 °C, the fracture process can be divided into three stages.

Stage I: When deformation occurs at relatively low temperatures (650–700 °C), as shown in Figure 15a, the experimental steels are located in the austenite + ferrite two-phase region. Because the deformation temperature is below the equilibrium transformation temperature, substantial undercooling occurs, thereby enhancing the driving force for the austenite-to-ferrite transformation. This results in a significant increase in the fraction of ferrite, leading to the formation of a thick and continuous ferrite layer, particularly along the prior austenite grain boundaries. The thicker ferrite layer can accommodate the strain mismatch between the austenite and ferrite phases, thereby effectively relieving local stress concentrations arising from differences in hardness and plasticity. Consequently, within this temperature range, the steel exhibits a relatively high RA and excellent hot ductility.

Stage II: As the deformation temperature rises to 800 °C, the transformation of austenite to ferrite is inhibited due to the reduced degree of undercooling, resulting in a decreased ferrite fraction, with only a network-like ferrite film forming along the austenite grain boundaries. This ferrite film exhibits low strength and poor ductility and is therefore prone to localized strain concentration under applied stress, thereby serving as a preferential nucleation site for microcracks. Consequently, the 0.09Sb steel reaches its minimum ductility temperature, while the hot ductility of the 0.15Sb steel also deteriorates significantly. Subsequently, when the deformation temperature increases to 900 °C and the material enters the single-phase austenite region, ferrite films no longer form at the grain boundaries. Instead, coarse precipitates such as Ti(C,N) and MnS preferentially precipitate at the grain boundaries, thereby promoting crack nucleation and reducing intergranular bonding strength; this accounts for the minimum ductility temperature observed in the 0.15Sb steel. Furthermore, as indicated by the non-equilibrium grain boundary segregation model and the quantitative EPMA point-scanning analysis (Figure 12 and Figure 14), this temperature range also results in severe non-equilibrium Sb grain boundary segregation, which further decreases grain boundary cohesion, weakens grain boundary fracture resistance, and increases the susceptibility to intergranular cracking. As shown in Figure 15b, Under the combined effects of grain boundary ferrite films, coarse precipitates, and non-equilibrium Sb grain boundary segregation, stress becomes highly concentrated at the grain boundaries, causing cracks to propagate along the grain boundaries and ultimately resulting in intergranular fracture. Macroscopically, this manifests as a significant reduction in the reduction in area and the formation of a hot ductility trough. Consequently, in industrial continuous casting processes, hot ductility can be improved by appropriately increasing the casting speed and elevating the temperature of the secondary cooling zone. Increasing the casting speed increases the strain rate, thereby improving the hot ductility of the cast billet, whereas elevating the temperature of the secondary cooling zone enables the process to avoid the minimum ductility temperature and remain as far as possible from the low-ductility region, thereby further enhancing hot ductility.

Stage III: When the deformation temperature is further increased to 1050–1200 °C, the experimental steels enter the fully austenitic single-phase region. Grain boundary ferrite completely disappears, thereby eliminating the strain incompatibility associated with the coexistence of two phases. Moreover, at elevated temperatures, atomic diffusion is enhanced and dynamic recrystallization is significantly promoted. As shown in Figure 8c, dynamic recrystallisation provides a sufficient driving force for grain boundary migration at high temperature and under applied strain. When the grain boundary migration rate exceeds the grain boundary slip rate, microcracks formed during deformation are confined along grain boundaries, thereby inhibiting crack accumulation and propagation and significantly enhancing the hot ductility of the steel. During deformation, recrystallization nucleates and grows within the prior austenite grains, forming fine, equiaxed microstructures. The resulting fine-grained structure exhibits excellent intrinsic plasticity and effectively impedes crack propagation. In addition, partial dissolution of precipitates at high temperatures promotes grain boundary purification. Consequently, as shown in Figure 15c, in this stage, the experimental steel recovers excellent plastic deformability, the RA increases markedly, and hot ductility is significantly improved.

## 5. Conclusions

In this study, the effect of Sb on the hot ductility of corrosion-resistant steel was systematically investigated over the deformation temperature range of 650–1200 °C. The relationship between the ductility trough and factors including ferrite transformation, precipitation behavior, and non-equilibrium grain boundary segregation of Sb was analyzed. The main conclusions are summarized as follows:

(1) Within the temperature range of 650–1200 °C, ductility troughs for the 0.09Sb and 0.15Sb steels were observed at 726–949 °C and 736–995 °C, respectively. The minimum ductility of the 0.09Sb steel occurred at 800 °C, corresponding to a RA of 42.47%, whereas that of the 0.15Sb steel occurred at 900 °C, with a RA of 43.29%. Increasing Sb content broadened the ductility trough temperature range and shifted the minimum ductility temperature to a higher value.

(2) In the austenite–ferrite two-phase region at 800 °C, the reduction in hot ductility is attributed to grain boundary stress concentration caused by the formation of network-like ferrite films and non-uniform stress distribution within the austenite, thereby reducing grain boundary fracture strength. In the single-phase austenite region at 900 °C, the deterioration in ductility of the 0.15Sb steel is associated with the precipitation of coarse Ti(C,N) and MnS particles at the grain boundaries, which promote crack initiation and result in intergranular fracture. Furthermore, the non-equilibrium grain boundary segregation model and EPMA point-scanning results indicate that Sb segregation occurs at the grain boundaries, further weakening grain boundary cohesion and promoting intergranular fracture.

(3) In the fully austenitic region, disappearance of grain boundary ferrite eliminated the stress mismatch associated with the coexistence of two phases. Furthermore, enhanced dynamic recrystallization resulted in the formation of fine, uniform equiaxed grains, and the fracture mode transitioned to transgranular fracture, thereby restoring hot ductility.

(4) Increasing Sb content delayed ferrite transformation and broadened the transformation temperature range. Moreover, both the magnitude of non-equilibrium grain boundary segregation of Sb and its peak temperature increased significantly. Consequently, the minimum ductility temperature of the corrosion-resistant steel shifted to a higher value.

## Figures and Tables

**Table 1:** Chemical composition of the corrosion-resistant alloy steel (wt. %).

Steel	C	Si	Mn	P	S	Al	N	O	Cr	Ni	Cu	Ti	Sb
0.09Sb	0.063	0.25	1.01	0.004	0.002	0.021	0.0015	0.0015	0.92	0.15	0.29	0.140	0.09
0.15Sb	0.061	0.25	0.99	0.005	0.003	0.025	0.0014	0.0015	0.90	0.16	0.29	0.145	0.15

**Table 2:** Data used for the calculation of non-equilibrium grain boundary segregation.

Parameter	Data	References
*E*_gb_/eV	0.33–1.76 × 10^−4^*T*	[39]
*k*/eV·K^−1^	8.617 × 10^−5^	
*E*_b_/eV	0.56	[40]
*E*_f_/eV	1.6	[35]
*β*/μm	80	[17]
*D*_c_/m^2^·s^−1^	5 × 10^−5^ × exp[−1.98/(*kT*)]	[41]
*D*_s_/m^2^·s^−1^	0.101 × exp[−2.76/(*kT*)]	[40]
*δ*	1700, 300	[42]
*d*/nm	1	[17]

**Table 3:** Sb concentrations at grain boundaries and within grains of the tested steel, as determined by EPMA point-scanning analysis.

Steel	1	2	3	4	5	6	7	8
0.09Sb-Matrix	0.105	0.083	0.115	0.098	0.072	0.086	0.102	0.099
0.09Sb-GB	0.203	0.205	0.258	0.225	0.218	0.193	0.242	0.217
0.15Sb-Matrix	0.168	0.162	0.148	0.140	0.157	0.151	0.139	0.165
0.15Sb-GB	0.424	0.465	0.472	0.464	0.482	0.413	0.453	0.420

## Data Availability

The original contributions presented in this study are included in the article. Further inquiries can be directed to the corresponding author.
